# Liver-on-a-Chip: Searching for a Balance Between Biomimetics and Functionality

**DOI:** 10.3390/bios16040191

**Published:** 2026-03-26

**Authors:** Anton Murashko, Daniil Golubchikov, Olga Smirnova, Konstantin Oleynichenko, Anastasia Nesterova, Massoud Vosough, Andrei Svistunov, Anastasia Shpichka, Peter Timashev

**Affiliations:** 1 Institute for Regenerative Medicine, Sechenov University, 119435 Moscow, Russia; murashko_a_v_1@staff.sechenov.ru (A.M.); golubchikov_d_o@staff.sechenov.ru (D.G.); smirnova_o_a_2@staff.sechenov.ru (O.S.); nesterova_a_m@staff.sechenov.ru (A.N.); 2 Faculty of Materials Science, Lomonosov Moscow State University, 119991 Moscow, Russia; oleynichenko_k_n@staff.sechenov.ru; 3 Department of Regenerative Medicine, Cell Science Research Center, Royan Institute for Stem Cell Biology and Technology, ACECR, Tehran 1665659911, Iran; masvos@royaninstitiute.org; 4 Experimental Cancer Medicine, Institution for Laboratory Medicine, Karolinska Institute, 171 77 Stockholm, Sweden; 5 World-Class Research Center “Digital Biodesign and Personalized Healthcare”, Sechenov University, 119435 Moscow, Russia; svistunov_a_a@staff.sechenov.ru; 6 Chemistry Department, Lomonosov Moscow State University, 119991 Moscow, Russia

**Keywords:** liver-on-a-chip, drug testing, liver, microfluidics, microphysiology system

## Abstract

One of the common issues in the R&D of new drugs is the failure of clinical trials caused by the species-specific inadequacy of animal models to assess drugs’ efficiency and safety. Therefore, systems like organ-on-a-chip and, particularly, liver-on-a-chip (LOC) can be an efficient tool for recapitulating in vivo-like human physiology at the microscale. This review focuses on discussing LOC design, emphasizing its architecture and validation to reveal the trends in searching for a balance between biomimetics and functionality. We found that the huge variety of already published models can be divided into five groups based on their configuration complexity: flat one-channel, flat two-channel, vertically stacked multilayered, hexagonal-patterned, and multi-well chips. While researchers attempt to recapitulate the liver’s histology and its functions in detail by increasing the complexity of devices’ architectonics, industrial companies prefer to promote more simple and flexible solutions. Thus, the LOC designs of the future require neglecting some liver characteristics to make them standardizable and sustainable, which could facilitate their introduction into the market and clinics.

## 1. Introduction

Despite the progress achieved, drug development remains a highly cost-intensive procedure. In accordance with a recent systematic review [1], it could cost up to 4.54 billion US dollars to launch a new molecular entity. A significant part of the expenses includes both drug synthesis and screening and preclinical trials. However, most drug candidates might fail in clinical trials due to low effectiveness and biosafety issues [2,3,4,5]. Therefore, the model systems commonly applied in the pharmaceutical industry cannot fully recapitulate the conditions required to predict possible outcomes [6,7].

Since the liver is one of the key players in drug metabolism, liver-mimicking models have the highest priority [8,9]. Previously, only static 2D systems (monolayers) including hepatocytes were used. Typically, they were presented using a micropatterned multi-well plate coated with various hydrogels (e.g., collagen [10], decellularized liver matrix (DLM) [11], fibronectin-coated chitosan [12]). Nevertheless, they do not allow for the reproduction of mechanotransduction [13,14] or mass transfer [15,16]. Moreover, such systems are rarely used to model liver diseases because they do not possess the appropriate metabolic activity due to their limited nutrition supply and low cell viability [17,18]. In some cases, they can be improved by co-culturing several cell types. For instance, hepatocyte spheroids co-cultured with stellate and non-parenchymal cells expressed a high CYP450 level, which was up to 6 times higher than that seen with only hepatocyte spheroids [19,20].

The rapid development of microfluidic technologies and tissue engineering has enabled the birth of a new concept—organ-on-a-chip and, particularly, liver-on-a-chip (LOC) [21,22]. One of the main hypotheses was that dynamic flow integration might not only modulate a cell’s state but also mimic blood and bile flow common for the native tissue. This concept has been successfully realized and described in many studies [23]. The assessment carried out by Ewart et al. [24] showed that LOC had 87% sensitivity and 100% specificity to 27 toxic and non-toxic substances that met the qualification guidelines, which are developed by the Innovation and Quality consortium and define parameters for qualifying preclinical models. Further analysis demonstrated that such a performance level could increase R&D productivity and generate more than 3 billion US dollars per year. While the number of LOC devices is constantly growing, it is essential to provide a framework of their typical architectonics and functional assessment. Thus, this review aims to become a guide on designing new biomimetic LOC devices, focusing on their configuration, validation, and applicability for further translation into clinics and the market.

This review distinguishes itself by analyzing the inherent trade-off between the biomimetic complexity and practical functionality across different LOC designs. By categorizing devices based on the structural complexity and comparing the divergent priorities of academia and industry, we provide a unique perspective on the path toward standardization and clinical translation, ultimately serving as a practical guide for designing next-generation LOC platforms.

## 2. From Anatomy to Functioning: How to Find a Focus

**Figure 1:**
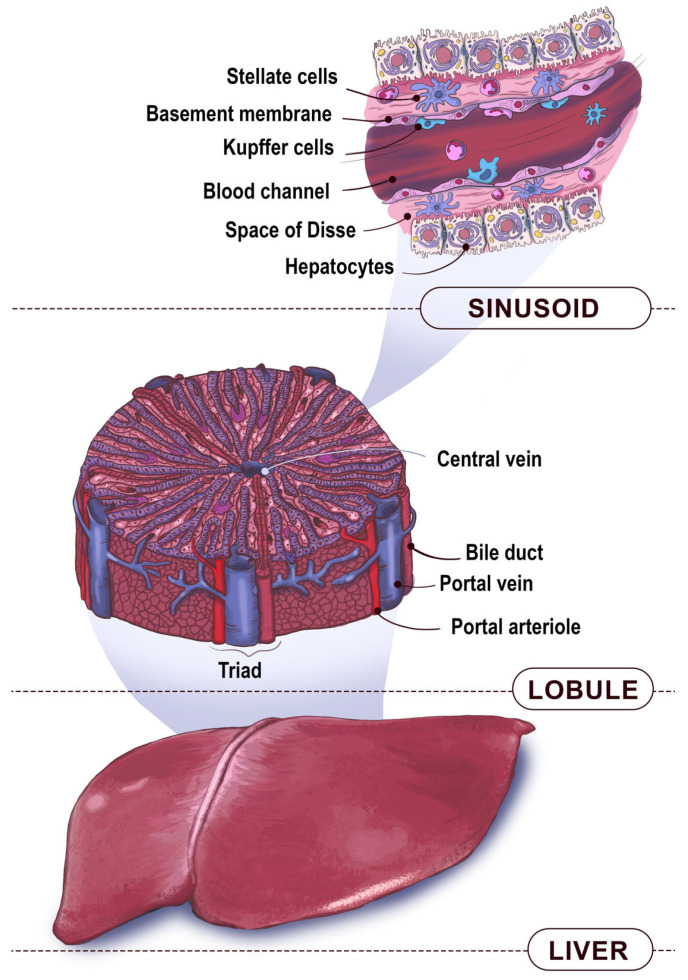
Three levels of the human liver anatomy: macroscopic (the body level), microscopic (the liver lobule level), and ultrascopic (the liver sinusoid structure).

Understanding of the liver’s functions may be based upon the morphological and metabolic zonation paradigms. The first one includes different levels, such as the macrostructure, microstructure, and ultrastructure (Figure 1). The macrostructure is important for processes at the body-size scale, such as physiological connections among several organs. The microstructure is a further descending level and a focus for histological analysis. The ultrastructure mostly refers to the cells’ inner structure and junctional complexes. Therefore, the macrostructure is the liver in whole; the microstructure contains the lobules, which are the smallest hexagonal-like liver units (0.9–1.1 mm^2^) and consist of hepatocytes (~10^6^ cells) and thousands of sinusoids fenestrated by capillaries; and the ultrastructure is mainly represented by the inner structure and cellular junctions (like those in the sinusoidal wall).

The sinusoid itself consists of four parts [25]. The first one is the vessel lumen and consists of blood and immune cells, such as Kupffer’s cells and lymphocytes. The second part is the endothelial barrier, formed by liver sinusoid endothelial cells (LSEC) with a discontinuous wall and fenestrated basal membranes. The third part is the Disse space, which contains hepatic stellate cells and blood plasma [26]. The last part is a cell layer consisting of hepatocytes and permeated by the bile ducts [27]. The sinusoid connects the lobule central vein to its corners, where three channels form a triad for fluid input into the lobule.

The liver’s function can also be described using the metabolic zonation paradigm based on oxygen and metabolite distribution within the sinusoid or the lobule. Here, there are three zone types [28]. The first one is located near the lobule corner and is notable for its high oxygen level, which enables processes such as beta-oxidation, gluconeogenesis, bile/cholesterol formation, and amino acid catabolism [29]. The second zone extends from the triad to the central vein and is described by the fluid dynamics. The third zone surrounds the central vein and is poorly perfused, which enables processes such as detoxication, drug biotransformation, ketogenesis, glycolysis, lipogenesis, glycogen synthesis, and glutamine formation [30].

Liver physiology focuses on its metabolism, which occurs within the paradigms described above. It secretes various metabolites and end products, influencing many functions within the organism. For instance, hepatocytes synthetize albumin supplied to the bloodstream (10–15 g per day). This controls the osmotic pressure and participates in drug transport. The liver inactivates ammonia formed from the extra nitrogen by transforming it into urea using five enzymes and a co-factor [31]. Moreover, it is involved in the drug metabolism, occurring in two phases. In Phase I, a substance is oxidized by the cytochrome P450 superfamily (CYP) enzymes [32]. According to Rendic et al., CYP3A4 has the highest contribution in drug metabolism (27%) and environmental and industrial chemicals (13%) [33]. Phase II involves the polar compound conjugation via various transferases: diphosphate (UDP)-glucuronosyltransferases, sulfotransferases, and glutathione S-transferases [34]. Thus, LOC should mimic the complex liver microenvironment and hierarchical cell organization that ensure its physiological functions (Figure 1).

## 3. From In Vivo to In Vitro: The Existing Models

### 3.1. Architectonics

**Figure 2:**
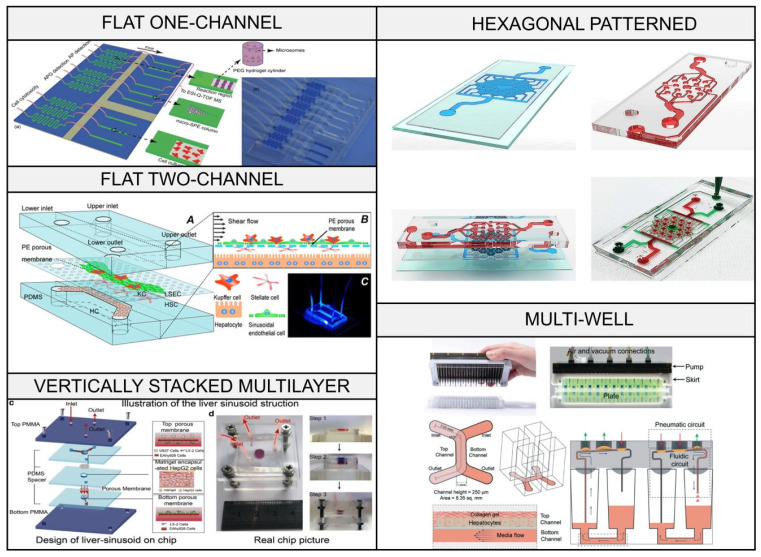
Typical LOC configurations: flat one-channel [35], flat two-channel [19], vertically stacked multilayer [36], hexagonal patterned [37], and multi-well [38] chips.

There are a lot of aspects that have to be taken into account to develop a platform mimicking the liver and its functions. A LOC is a liver-like device created by using achievements in various fields such as microfluidics, computer modeling, cell biology, etc. To date, an immense variety of LOC designs have been described in papers and introduced to the market. Each of them has its own features, determined by the inner architecture, fabrication techniques, cell types, etc. (Figure 2). Nevertheless, they could be classified into the following groups based on their configuration complexity: flat one-channel, flat two-channel, vertically stacked multilayered, hexagonal patterned, and multi-well chips (Table 1).

#### 3.1.1. Flat One-Channel Chips

A flat one-channel chip is the simplest perfused system in widespread use and basically consists of one channel with an inlet and an outlet [42]. The main benefit of such chips is the ease of their assembly and handling; therefore, most studies apply them to analyze the flow dynamics using different parameters [35,43,64]. Despite their relative simplicity, flat one-channel chips might have diverse designs and be fabricated using different techniques. For instance, a system presented by Kamei et al. [43,64] had a straight channel replicated into PDMS using a 3D printed mold. Mao et al. [35] fabricated a device with separated zigzag-like channels using soft lithography and prototyping.

Flat one-channel chips can be loaded with cells using two main approaches: cell perfusion and integration of the immobilized cells. In the first case, researchers mostly focus on fabricating liver spheroids or organoids within a chip. For instance, Lee at al. [65] formed a silicone microwell platform with semi-spherical cavities with a diameter of 500 µm and full height of 1000 µm that can potentially be further upgraded into a perfused chamber. A similar concept based on special cavities was realized in a paper by Lee et al. [41]. They formed a two-chamber perfusable system in which hepatocytes were seeded into rectangle-shaped cavities. Another method was demonstrated by Jun Ye Ong et al. [44], who used a bubble-like channel to immobilize the perfused cell spheroids and separated it from the flow using a micropillar array. Nevertheless, in the second case, researchers could fabricate more complex structures. In particular, Cui et al. [39] developed a chip containing a five-layer hexagonal construct formed by cells encapsulated within gelatin methacrylate (GelMA) and poly(ethylene) glycol diacrylate (PEGDA). Moreover, to realize this approach, novel techniques such as bioprinting can be applied, as was successfully shown by Bhise et al. [40].

In most cases, such chips imitate blood vessels within the liver [42,43,64], particularly the sinusoid [35]. However, Gori et al. upgraded a simple 1-channel model by adding microchannels to reproduce the endothelial barrier [66]. Nevertheless, one-channel chips fail to reproduce the liver zonation and other structures.

#### 3.1.2. Flat Two-Channel Chips

One of the first concepts to increase chip complexity was based on the addition of a second channel to improve the flow dynamics and reproduce four parts of the sinusoid [19,46,47,48,49,51]. For instance, to study the communication between injured hepatocytes and stellate cells, Zhou et al. [48] constructed a device composed of two chambers divided by a retractable wall. However, the most common design includes two chambers separated by a membrane (with 8 µm pores) and perfused using an upper channel [19,47]. Typically, human umbilical vein endothelial cells (HUVEC or LSEC) and hepatocytes are seeded at the top and at the bottom (on a membrane or a chamber bottom), respectively. Together with monolayers, 3D cell constructs can be applied to a lower chamber [49,50]. Hydrogel-embedded cells are claimed to require low cell concentrations and medium volumes for perfusion (24 mL or 1 mL per day for dynamic and static conditions, respectively). However, there are models where such constructs are only placed into an upper chamber that mimics the bile channel [51]. Moreover, Deng et al. [46] fabricated a LOC device containing two lateral vacant channels with a central cell-loaded chamber separated with a barrier array. A similar design can be applied to reproduce the crosstalk between the liver and other organs. For instance, to model hepatic steatosis, Jeon et al. [67], inspired by Chen et al. [68], fabricated a gut–liver-on-a-chip consisting of three PDMS layers (top—a medium reservoir; middle—a Caco-2 cell monolayer; bottom—a HepG2 cell monolayer). As it was gravity-driven, there was no need to apply a pumping system. Flat two-channel chips could also reproduce the nutrients’ mass transfer through the Disse space, avoiding the flow-induced shear stress. Such a concept was realized by Meng et al. [52], who formed two tree-like channels separated by a hydrogel barrier and seeded with hepatocytes’ spheroids (out) and endothelial cells (in).

#### 3.1.3. Vertically Stacked Multilayered Chips

To improve the LOC’s functions, researchers have proposed adding 3D vertical interconnections. Typically, such chips aim to mimic channels crossing the lobule [15,16]. For instance, Du et al. formed a vertically stacked multilayered device consisting of a bottom outlet—the central vein—and hepatic artery and portal vein channels connected to a hexagonal chamber [56].

This type of chip enables the radial flow regime. This was realized by Deng et al. [36], who formed a device consisting of two PMMA substrates with three PDMS layers and a channel crossing them and containing two membranes and Matrigel-embedded HepG2 cells in between. The top layer included a narrow channel imitating the sinusoid. The inlet flow rate was 1 µL per min, similar to that in vivo. In the cell-containing zone, the computing simulation showed the radial flow typically used to reproduce the in vivo fluid distribution [69,70,71]. Nevertheless, flow regimes in such chips are highly limited.

#### 3.1.4. Hexagonal Patterned Chips

Hexagonal patterned chips aim to provide more possibilities in adjusting the flow parameters. For instance, Lorente et al. [72] showed that the dendritic-based architecture can provide a higher benefit than the radial fluid distribution. The calculations performed are of great value, particularly in explaining the fluid distribution inside a chip, as offered by Banaeiyan et al. [37]. Their device, consisting of two layers, had the radial fluid distribution in a hexagonal chamber and the dendritic one in channel branches.

The main chamber in such chips can reproduce the lobule structure highly efficiently. For example, Ma et al. [59] fabricated a lobule-like microtissue formed by four layers: a fluidic one including a cell culture chamber, a control one containing a pneumatic system, a thin PDMS one with adhesive and supporting functions, and a bottom glass one. The first layer was hexagonal and had a 24-pillar array (square ones to mimic a space among the adjacent lobules and cylindrical ones to provide a mechanical support).

#### 3.1.5. Multi-Well Chips

Multi-well chips (perfusable microbioreactors) can be considered the highest step in design development due to their high reproducibility, variety of cells, flow regimes used, etc. Such a chip was offered by Weng et al. [62] and inspired by the lattice growth mechanisms from material sciences. They placed cell spheroids into a hexagonal non-structured space with a micro-patterned hydrophobic PDMS membrane, pressure inlets in its corners, and outlets in its center, which created radial flow distribution with an increasing spread rate.

One of the most interesting systems published was developed by Domansky et al. [61]. They fabricated a multi-well chip to monitor the oxygen transport and consumption in primary liver cell cultures. It consisted of two parallel arrays with seven wells in each, formed by two chambers divided with a wall. There was a space under them with a medium flowing through their volume. In one of the chambers, there was a cell-seeded scaffold presented by a circular extracellular matrix (ECM)-coated polymer wafer with a 769-microchannel array equipped with a 5 µm filter and a filter support.

### 3.2. Functionality Assessment

As LOC can be applied to study various issues (drug testing, cell biology, disease modeling, etc.), researchers use different methods to assess their functionality. Nevertheless, most of them enable evaluating the albumin and urea synthesis, the oxygen consumption and distribution, and the CYP activity.

To analyze the albumin expression, both qualitative and quantitative methods were used. In the first group, PCR was applied [51,55,57]; however, the protein’s presence could not be proven. Therefore, immunocytochemical staining became a method of choice [43,57,64,66]. In the second group, the most common assays are enzyme-linked immunosorbent (ELISA) [19,37,40,51,52,56,57,58,60,62], colorimetric [36,46], and multiplex [38] assays which show the albumin concentration in a supernatant.

The urea concentration in LOC can be measured using both ELISA [58] and colorimetric [19,37,39,51,52,56,57] methods.

CYP3A4 and CYP1A2 are commonly used as markers to assess the hepatocyte’s metabolic activity within LOC devices. To show their expression, authors prefer to apply PCR and immunostaining [41,47,51,55,57] as well as ELISA [58]. Nevertheless, the CYP activity can be proven only by methods based on reactions which it catalyzes. These can be divided into two groups: those based on luminogenic substrates [38,56,62] and those based on the concentration of end- and by-products [19,46,47,52]. The second group includes spectrophotometry and liquid chromatography–mass spectrometry (LC-MS).

In most cases described above, commercial kits are available that make the analysis easy and reproducible.

#### 3.2.1. Urea

Flat one-channel chips ensure an increase in the urea concentration [41,65]. However, in some models, the difference between the static and dynamic conditions is almost insignificant. As a control, authors usually use a cell culture cultivated in a Petri dish.

Findings reported for the two-channel chips are controversial. On the one hand, many researchers have shown that the use of such chips causes a rise in the urea concentration [46,51,52] depending on the flow. For example, a system by Rennert et al. maintained a stable urea level over 96 h; however, under static conditions, the concentration decreased by 20%, similar to findings for a culture in a Petri dish [47]. On the other hand, Do et al. revealed a slight decrease in the urea concentration [19].

Vertically stacked multilayered chips showed a positive urea concentration trend. Compared to a control, the value could be two times higher (or even more) [36,56]. The trend was close to being linear in [56], while the value remained stable with a noise deviation in [36].

Hexagonal patterned chips also demonstrated either a stable value or a positive trend in the urea concentration that was time-dependent [37,57,58,60]. A chip described in [37] enabled stable urea concentration with a random increase; however, outliers in the data without an explicit tendency require special attention.

The number of multi-well chips is limited, and the urea concentration was measured in a paper by Weng et al. They revealed that, during seven days experiment, the proposed design was holding the increased urea concentration level. There is no data on the urea concentration for the first 7 days, making it impossible to analyze its trend [62].

#### 3.2.2. Albumin

The albumin concentration is measured in most papers reviewed. Compared to a control, flat two-channel chips demonstrated an increased protein level. Typically, this level remains stable or grows with time [19,46,52]. For instance, Rennert et al. revealed that a non-perfused chip showed a higher albumin concentration than that in a control, but the trend was negative; when it was perfused, the protein level rose over time [47]. In a paper by Lee et al., the albumin concentration in a chip was increased and did not change significantly; however, on the last day, it dropped [51].

Vertically stacked multilayered chips showed similar trends. Do et al. revealed that, while the urea concentration remained similar, the albumin concentration grew for 7 days [56].

Hexagonal patterned chips showed an upward trend with a constant growth rate. Chips described by Janani et al. and Ya et al. enabled an increase in the albumin concentration for 14–16 days that exceeded that of any device with a less complex architecture [57,58]. The albumin concentration was reported to depend on the shear stress [58]. Liu et al. demonstrated stable growth for 8 days and its dependency on the glucose level (under low glucose conditions, the albumin concentration rose and in 3 days reached the value obtained under high glucose conditions) [60]. Banaeiyan et al. fabricated a chip enabling a rise in the protein level for 13 days (3.5–4 times higher than that in a control), which remained stable for the following 7 days [37].

Multi-well chips can be considered to ensure a high protein secretion level similar to that in the hexagonal patterned ones. In particular, Weng et al. revealed an increased albumin concentration, growing for the first 7 days and becoming equal to that in the control on day 14 [62]. However, a chip designed by Tan et al. showed a stable protein level for 13 days, exceeding that of a static control [38].

#### 3.2.3. Oxygen

The oxygen concentration and distribution could reflect the hepatocytes’ state and activity, as it plays a crucial role in many processes such as gene expression, cell differentiation, etc. [54,58,73,74]. Nevertheless, the number of studies evaluating these parameters is limited.

Even a simple one-channel model created by Ghafoort et al. allows the oxygen gradient influence to be studied [75]. The authors serially connected four microfluidic chips to form a system of eight interconnected chambers and revealed the correlation between the albumin expression and oxygen level in cells along the liver acinus. A more complex one-channel model was described by Kwon et al. to analyze the drug-induced zonal hepatoxicity [76]. They imitated the liver acinus by using two wells mimicking the periportal and perivenous zones and the lobule by arranging cells radially. This chip’s design enabled the oxygen gradient within the acinus to be reproduced. A two-channel chip created by Rennert et al. showed a downward oxygen saturation trend depending on time [47]. A vertically stacked multilayered chip developed by Bavli et al. allowed one to study the oxygen concentration after adding drugs and revealed its increase [55].

There is a lack of data on assessing the oxygen level in multi-well chips. However, Domansky et al. measured it and revealed that oxygen dissolved in a medium flow at a high rate could not be utilized by cells [61]. Moreover, Bushe et al. developed a system enabling the estimation of the oxygen consumption and showed the time-dependent downward trend in the oxygen concentration [63].

#### 3.2.4. Cytochrome Enzymes

CYP expressed by hepatocytes is responsible for the drug’s transformation in the organism, and assessment of its activity is widely used in functional assays and defines further LOC applications [77,78,79].

Flat one-channel chips can be tested to reveal the CYP activity. For instance, Zheng et al. [80] developed a one-channel device with microcavities filled with spheroids from hepatocytes and HUVEC and analyzed the acetaminophen (APAP) and mitomycin C transformation catalyzed by CYP1A2 and CYP3A4 [81]. However, even when being perfused, the designed chip did not enable high CYP activity significantly exceeding a static control. Nevertheless, there is a lack of chips with such an architecture, allowing for the study of the drug’s hepatotoxicity.

Two-channel chips typically ensure a higher CYP450 expression level than static systems. In particular, under perfused conditions, Rennert et al. detected that the stable CYP3A4 expression increased by 20–25%, causing improved midazolam metabolism [47]. As further analysis showed, the CYP1A2 and CYP2E1 expression levels rose in the perfused two-channel chips [19,52] used to study the APAP- and dextromethorphan-induced hepatotoxicity. Interestingly, such chips might prolong CYP activity for up to 21 days compared to a multi-well plate [53], and were shown to be more sensitive (IC50 value).

Vertically stacked multilayered chips are superior to two-channel ones due to a more complex channel architecture. Nevertheless, there is a lack of studies using such chips and assessing the CYP activity. For instance, Du et al. revealed that the CYP1A2 and CYP3A4 activity increased for 7 days in their LOC device; however, the period was insufficient for comparison with other studies and showed a further significant drop in CYP activity. Moreover, while modeling non-alcoholic fatty liver disease using the designed chip, the authors revealed a decrease in CYP activity by 10–25% [56]. Bavli et al. demonstrated the applicability of such chips to study the mitochondrial dysfunction induced by rotenone and troglitazone: the CYP expression levels were higher than those under static conditions [55,82].

Due to the radial flow distribution being similar to that in vivo, hexagonal patterned chips can be used to demonstrate increased CYP activity for 14 days (especially at high flow rates) and its dependence on the oxygen distribution [58]. Additionally, Janani et al. revealed rapid CYP activity increment over 5 days followed by minor fluctuations for the next 10 days [57]. Nevertheless, the common trends in the APAP hepatotoxicity assay are similar to those for flat two-channel chips.

Multi-well chips are of particular interest due to their high reproducibility [83]. Such a chip described by Tan et al. demonstrated CYP-level decrement in 7 days that was similar to that observed in a system designed by Delalat et al. [38,53]. The maintenance of the cell metabolic activity in hexagonal multi-well chips was shown to be improved and remained stable between day 7 and day 14. The APAP hepatotoxicity was revealed to be higher in Zone II, which might be caused by the decreased oxygen concentration and therefore the enhanced CYP activity [58,62].

## 4. Challenges and Prospects

**Figure 3:**
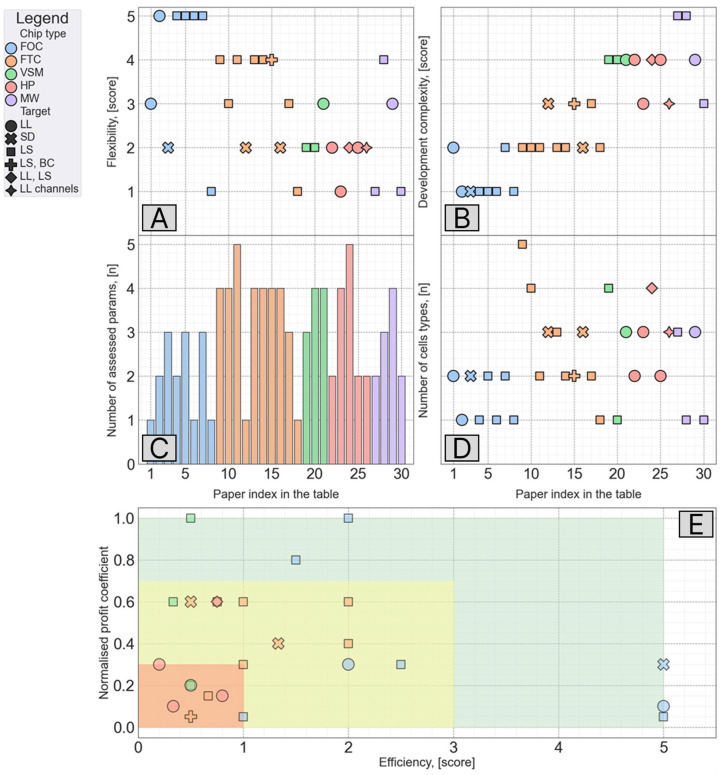
Parametric analysis of LOC devices (Table 1). The analysis included 30 papers described in Table 1 and divided into 4 groups depending on the LOC configuration complexity (flat one-channel (FOC), flat two-channel (FTC), hexagonal patterned (HP), vertically stacked multilayered (VSM), and multi-well (MW) chips). The search was performed via Scopus and PubMed databases using keywords such as “liver-on-a-chip”, “microfluidics”, “microphysiology system”, “lab-on-a-chip”, “liver”, etc. Reviews, letters, and editorials were excluded as well as papers describing an enzyme-immobilized models or containing only numerical simulations (without cells). The legend also includes the target part: BC—bile channel; LL—liver lobule; LS—liver sinusoid; SD—Disse space. The LOC comparison was performed using the following parameters: flexibility, complexity, number of assessed parameters, number of cell types, and profit-efficiency correlation. Each of them was defined as follows: (**A**) Flexibility—score (1–5); adaptability of a system to alternative research endeavors with a focus on the measurement of different parameters or the reproduction of distinct liver features. (**B**) Complexity—score (1–5); complexity of the chip’s configuration and its fabrication. (**C**) Number of assessed parameters—score (1–5); number of parameters measured to show the functionality of the developed LOC system: albumin expression, urea concentration, oxygen concentration, CYP450 expression, and others. (**D**) Number of cell types—number of cell types used to fabricate a LOC system. (**E**) Profit-efficiency correlation—profit coefficient is the number of measurable parameters and represented cells (used to assess the overall comprehensiveness of a research); efficiency coefficient is the ratio of flexibility to complexity (used to evaluate the chip’s pertinence for future research, specifically assessing the ease of assembling a highly capable system). Limitation. The parameters assessed using a score scale are subjective. To alleviate this issue, the score values were given by three independent researchers and used as a mean value.

The further development of LOC systems is closely linked to the body-on-a-chip concept. Its framework elicits significant interest, and particular attempts have been realized, e.g., by Lee et al. [84] and Skardal et al. [85]. Researchers have tried to find a balance between biomimetics and functionality. To reveal these trends, we performed a LOC parametric analysis (Figure 3) using the following criteria: flexibility, complexity, system parameters to be measured, cell types used, and profit-efficiency correlation. Figure 3 displays a discernible negative trend across the papers analyzed, while Figure 3 exhibits a contrasting positive trend. The synthesis of these two trends yielded a significant inverse correlation between the notions of chips’ flexibility and complexity. Notably, the number of parameters exhibited a consistent pattern from one paper to another, with a tendency towards simplicity observed in flat one- and two-channel chips, which featured a diminished minimum parameter count. The analysis also revealed that typically researchers used two cell types to fabricate a LOC system. We attempted to elucidate the “efficiency” and “profit coefficient” of these chips by establishing a conceptual link between these two heuristic parameters (Figure 3). Furthermore, we tried to discern distinguishable clusters based on chips’ configuration. Both the DBSCAN and spectral clustering algorithms yielded clustering scores lower than 0.5. The subjective evaluation of the dependency indicated that the hexagonal-patterned chips exhibited suboptimal quality due to their elevated complexity coupled with limited flexibility, resulting in a reduced number of discernible cells with quantifiable parameters. Conversely, flat one-channel chips displayed a consistent pattern (one of them achieved the highest profit score). In contrast, the vertically stacked configuration, while characterized by heightened complexity and diminished flexibility, yielded intriguing findings, resulting in an average score in the proximity of 0.6.

Interestingly, commercially available chips usually have simple architectonics and are mostly not organ specific. In 2017, Lee-Montiel et al. [86] demonstrated the possibility of fabricating the Liver Acinus MicroPhysiology System (LAMPS) using a single chamber chip produced by Nortis (USA). Two-channel chips are also widely applied; the devices by Micronit, Emulate, and HemoShear Therapeutics (including their modifications) in particular have shown their applicability in mimicking the liver functions [87,88,89,90]. Nevertheless, the most common type is a multi-well array [38]. The simplicity and flexibility of commercial chips is claimed to be a key to the data reproducibility and their easy handling; however, the results published showed high variability and loss of interactions.

The discrepancies in the urea and albumin concentrations, as well as in the CYP activity between different LOC architectures, highlight a critical need for deeper investigation. While our results demonstrate variability, attributing these differences remains challenging. Factors such as the shear stress ranges across varying chip designs likely contribute significantly; differing flow rates can alter the cellular behavior and metabolic function. Furthermore, variations in the detection methods employed by different research groups—and the inherent subjectivity of scoring parameters [91,92,93,94]—may introduce a bias. The materials used for chip fabrication could also play a role, influencing cell adhesion, differentiation and ultimately functional output. The potential increase in the complexity and biomimetics of commercial LOC devices and the applicability of existing studies are strongly associated with issues including the industrial translation and automation of techniques based on bioprinting, organoid formation and culturing, laser-assisted additive technologies, etc., to fabricate them; analytical methods’ adaptation to assess results in situ, e.g., cell tracers, probes, in situ PCR [95,96,97]; and high-throughput screening software platforms [98]. These challenges underscore the importance of standardized protocols for LOC fabrication, operation, and analysis to improve reproducibility and comparability across studies.

Nevertheless, besides the technological challenges mentioned above, there are also those to be answered mostly by scientists. First, the multi-organ system design may inadvertently limit representation of some processes (pathological and physiological reactions, drug absorption, metabolism, excretion, etc.) resulting in data loss or their inapplicability. Moreover, the inherent variability does not permit a full understanding of the effects observed in the organism, showing the need to establish a well-defined and highly reproducible lower-level framework which can serve as a base for further modeling.

It is worth mentioning that the field of LOC applications is constantly expanding. If the first models mostly reproduced normal tissue, now one of the main focuses is disease modeling [99,100,101,102,103,104]. In particular, Naworth et al. [105] proposed a flat two-channel chip for modeling alcohol-associated liver disease; to model nonalcoholic fatty liver disease (NAFLD), Wang et al. [106] fabricated a perfused chamber where liver organoids are exposed to free fatty acids. Moreover, recent research has shown the LOC applicability and efficiency of studying virus-induced liver diseases, which could improve the analysis quality [28,107]. For instance, microfluidic chips containing hepatocytes, T-cells, and Kupffer cells enable an enhanced immune response against hepatitis B (HBV) and C (HCV) viruses to be revealed [108,109]. Even relatively simple one-channel and two-channel chips can be successfully used as a platform to model HBV infection [109,110,111,112]. A significant driver for LOC development is the acknowledged inadequacy of traditional animal models in predicting the human drug response due to species-specific differences in the hepatic metabolism and immune responses [113,114]. While animal testing remains essential in pre-clinical studies, ethical concerns and regulations are increasingly strengthening restrictions on its use. LOC technology offers a compelling alternative by utilizing human cells in vitro, potentially providing more physiologically relevant data. LOC applications are not limited by studying only hepatotropic infections; they were shown to be an efficient tool to discover virus-induced hepatic injuries caused by SARS-CoV-2 [115,116,117]. Furthermore, researchers have developed LOC models to mimic fatty liver disease, allowing investigation of the underlying mechanisms and potential therapeutics [98]. Advances also include chips designed to replicate metastasis in kidney cancer progressing to the liver, aiding in treatment efficacy prediction [99]. However, the pursuit of complete biomimicry may hinder standardization and scalability—critical factors for widespread adoption in preclinical drug testing. As such, future LOC designs may benefit from strategic prioritization of the key hepatic functions over the exhaustive morphological replication. While detailed modeling of the liver’s complex architecture is valuable, commercial viability often necessitates simplification. This requirement suggests focusing on essential elements like hepatocyte function, bile canaliculi representation for toxicity studies, and perfusion to mimic systemic circulation. The inclusion of immune cell interactions, as demonstrated by studies modeling HBV/HCV infection [108,109], represents a high-value feature; at the same time, replicating the full spectrum of hepatic immune responses may be less critical in initial screening assays. Ultimately, neglecting certain aspects of the liver physiology—such as complete zonal heterogeneity or complex innervation—could allow for more robust and reproducible LOC platforms suitable for large-scale pharmaceutical applications.

This development highlights the LOC’s potential to recapitulate complex biomechanical environments relevant to fibrosis and other liver pathologies. The further development of this direction is expected to significantly impact the R&D of antivirals in the pharmaceutical industry.

## 5. Conclusions

LOC devices have shown their high potential as efficient tools to model the liver and its diseases. Despite the wide variety of chips described, there is no single concept that could specify the required liver functions and ensure reliable and reproducible results. While scientists aim to maximally reproduce the liver by increasing the LOC complexity, commercial companies have chosen simplicity and flexibility. Thus, LOC designs of the future will require some features in the liver morphology and physiology to be neglected in order for them to be standardized, introduced onto the market, and applied in preclinical trials.

## Figures and Tables

**Table 1:** Liver-on-a-chip systems: fabrication and functionality assessment. The table provides an overview of studies on microfluidic-based approaches. The “Functionality Assessment” column indicates the types of evaluations performed in each study. A plus sign (+) means the parameter was measured, while a minus sign (−) indicates it was not.

Reference	Target Structure	Chip		Cells		Functionality Assessment				
		Material	Fabrication Method	Types	Fabrication Method	Urea	Albumin	Oxygen	CYP	Others
Flat one-channel chips										
Cui et al. [39]	LL	PDMS, PEGDA, GelMA	NA, PP	HepG2, HUVEC	HEC	+	−	−	−	−
Bhise et al. [40]	NA	PDMS, GelMA	LC, BP	HepG2/C3A	CS, HEC	−	+	−	−	+
Lee et al. [41]	NA	PDMS	SL	PH, HSC	CS	+	+	−	+	−
Kulsharova et al. [42]	NA	PDMS, PC, COC	SL, NA	Huh7	CL	+	+	−	−	−
Kamei et al. [43]	NA	PDMS	3DP	HepG2, hiPSC	CL	−	+	−	+	+
Mao et al. [35]	NA	PDMS, PEGDA	SL	HepG2	CL	−	−	−	−	+
Jun Ye Ong et al. [44]	NA	PDMS	SL	HepaRG, PH	CS	+	+	−	+	−
Matsumoto et al. [45]	NA	PDMS, glass	SL	HepG2	CL	−	−	+	−	−
Flat two-channel chips										
Du et al. [19]	LS	PDMS	SL	PH, LSEC, KC, HSC, NPC	CL	+	+	−	+	+
Deng et al. [46]	LS	PDMS	SL	HepG2, LX-2, HUVEC, U937	HEC	+	+	−	+	+
Rennert et al. [47]	LS	COC	MM	HepaRG, HUVEC	LO	+	+	+	+	+
Zhou et al. [48]	NA	PDMS	SL	Rat PH, LX-2	CL	−	−	−	−	+
Prodanov et al. [49]	LS	PDMS	SL	PH, EA.hy926, LX-2	HEC	+	+	−	+	+
Hegde et al. [50]	NA	PDMS	SL	Rat PH	HEC	+	+	−	+	+
Lee et al. [51]	LS, BC	PEVA, gelatin, ECM	3DP, BP	HepaRG, HUVEC	HEC	+	+	−	+	+
Meng et al. [52]	SD	PDMS, GelMA	3DP	HepG2, HUVEC, LX-2	CS, HEC	+	+	−	+	+
Delalat et al. [53]	NA	PDMS	SL	Rat PH	CL	+	+	−	+	−
Moya et al. [54]	LS	PMMA	NA, 3DP	PH	CL	−	−	+	−	−
Vertically stacked multilayered chips										
Deng et al. [36]	LS	PDMS	SL	HepG2, LX2, EAhy926, U937	HEC	+	+	−	−	+
Bavli et al. [55]	LS	PMMA, PDMS	CNC, LC	HepG2/C3A	HEC	−	+	+	+	+
Du et al. [56]	LL	PDMS	SL	HepaRG, LX-2, LSEC	HEC	+	+	−	+	+
Hexagonal patterned chips										
Banaeiyan et al. [37]	LL	PDMS	SL	HepG2, hiPSC-d-HC	CL	+	+	−	−	−
Janani et al. [57]	LL	ECM	BP	ADMSC-d-HC, HSC, HUVEC	HEC	+	+	−	+	+
Ya et al. [58]	LL, LS	PDMS, PMMA	SL	PH, LSEC, HSC, KC	HEC	+	+	+	+	+
Ma et al. [59]	LL	PDMS	SL	HepG2, HAEC	CL, HEC	−	−	−	+	+
Liu et al. [60]	LL channels	PMMA	NA	PH, HSC, LSEC	CL, HEC	+	+	−	−	−
Multi-well chips										
Domansky et al. [61]	NA	PC, PS, PU, collagen	CNC	Rat HC, LSEC	CL	+	−	+	−	−
Tan et al. [38]	NA	PC, PI, COC, FEP	LC	PH	HEC	−	+	−	+	+
Weng et al. [62]	LL	PDMS	SL	Rat PH, HSC	CL	+	+	−	+	+
Busche et al. [63]	LS	PS	CNC	PH	CL	−	−	+	−	+

## Data Availability

No new data were created or analyzed in this study.

## Abbreviations

The following abbreviations are used in this manuscript:

APAP—acetaminophen; CYP—cytochrome; DLM—decellularized liver matrix; ECM—extracellular matrix; ELISA—enzyme-linked immunosorbent assay; HBV—hepatitis B virus; HCV—hepatitis C virus; HUVEC—human umbilical vein endothelial cells; GelMA—gelatin methacrylate; LOC—liver-on-a-chip; LAMPS—liver acinus microphysiology system; LC-MS—liquid chromatography–mass spectrometry; LSEC—liver sinusoidal endothelial cells; NAFLD—nonalcoholic fatty liver disease; FEP—fluorinated ethylene propylene; GelMA—gelatin methacrylate; PC—polycarbonate, PDMS—polydimethylsiloxane; PEGDA—poly(ethylene) glycol diacrylate; PI—polyimide; PMMA—poly(methyl) methacrylate; PS—polystyrene; PU—polyurethane; BC—bile channel; LL—liver lobule; LS—liver sinusoid; SD—Disse space; COC—cyclic olefin co-polymers; ECM—extracellular matrix; 3DP—3D printing; BP—3D bioprinting; CNC—computer numerical control machining; LC—laser-cut; MM—metals mechanical machining; SL—soft lithography; CL—cell layers, CS—cell spheroids, HEC—hydrogel-embedded cells; LO—liver organoids; NA—no data available; FOC—flat one-channel chips; FTC—flat two-channel chips; HP—hexagonal patterned chips; MW—multi-well chips; VSM—vertically stacked multilayered chips.

## References

[1] Schlander M., Hernandez-Villafuerte K., Cheng C.-Y., Mestre-Ferrandiz J., Baumann M. (2021). How Much Does It Cost to Research and Develop a New Drug? A Systematic Review and Assessment. PharmacoEconomics.

[2] Luo X.-Y., Wu K.-M., He X.-X. (2021). Advances in drug development for hepatocellular carcinoma: Clinical trials and potential therapeutic targets. J. Exp. Clin. Cancer Res..

[3] Wouters O.J., McKee M., Luyten J. (2020). Estimated Research and Development Investment Needed to Bring a New Medicine to Market, 2009–2018. JAMA.

[4] Huang L.-K., Chao S.-P., Hu C.-J. (2020). Clinical trials of new drugs for Alzheimer disease. J. Biomed. Sci..

[5] Ratziu V., Friedman S.L. (2023). Why Do So Many Nonalcoholic Steatohepatitis Trials Fail?. Gastroenterology.

[6] Golding H., Khurana S., Zaitseva M. (2018). What Is the Predictive Value of Animal Models for Vaccine Efficacy in Humans?: The Importance of Bridging Studies and Species-Independent Correlates of Protection. Cold Spring Harb. Perspect. Biol..

[7] Franco R., Cedazo-Minguez A. (2014). Successful therapies for Alzheimer’s disease: Why so many in animal models and none in humans?. Front. Pharmacol..

[8] Dirven H., Vist G.E., Bandhakavi S., Mehta J., Fitch S.E., Pound P., Ram R., Kincaid B., Leenaars C.H.C., Chen M. (2021). Performance of preclinical models in predicting drug-induced liver injury in humans: A systematic review. Sci. Rep..

[9] Baudy A.R., Otieno M.A., Hewitt P., Gan J., Roth A., Keller D., Sura R., Van Vleet T.R., Proctor W.R. (2020). Liver microphysiological systems development guidelines for safety risk assessment in the pharmaceutical industry. Lab A Chip.

[10] Khetani S.R., Bhatia S.N. (2008). Microscale culture of human liver cells for drug development. Nat. Biotechnol..

[11] Zhu X., Wu Q., He Y., Gao M., Li Y., Peng W., Li S., Liu Y., Zhang R., Bao J. (2022). Fabrication of Size-Controllable and Arrangement-Orderly HepG2 Spheroids for Drug Screening via Decellularized Liver Matrix-Derived Micropattern Array Chips. ACS Omega.

[12] Rajendran D., Hussain A., Yip D., Parekh A., Shrirao A., Cho C.H. (2017). Long-term liver-specific functions of hepatocytes in electrospun chitosan nanofiber scaffolds coated with fibronectin. J. Biomed. Mater. Res..

[13] Shu X., Li N., Wu Y., Li W., Zhang X., Li P., Lü D., Lü S., Long M. (2021). Mechanotransduction of liver sinusoidal endothelial cells under varied mechanical stimuli. Acta Mech. Sin..

[14] Mitten E.K., Baffy G. (2022). Mechanotransduction in the pathogenesis of non-alcoholic fatty liver disease. J. Hepatol..

[15] Yang H., Sun L., Pang Y., Hu D., Xu H., Mao S., Peng W., Wang Y., Xu Y., Zheng Y.-C. (2021). Three-dimensional bioprinted hepatorganoids prolong survival of mice with liver failure. Gut.

[16] Narendran G., Walunj A., Kumar A.M., Jeyachandran P., Awwad N.S., Ibrahium H.A., Gorji M.R., Perumal D.A. (2023). Experimental Demonstration of Compact Polymer Mass Transfer Device Manufactured by Additive Manufacturing with Hydrogel Integration to Bio-Mimic the Liver Functions. Bioengineering.

[17] Cottier K.E., Bhalerao D., Lewis C., Gaffney J., Heyward S.A. (2023). Micropatterned primary hepatocyte co-culture (HEPATOPAC) for fatty liver disease modeling and drug screening. Sci. Rep..

[18] Choi S.H., Fukuda O., Sakoda A., Sakai Y. (2004). *Enhanced cytochrome* P450 capacities of Caco-2 and Hep G2 cells in new coculture system under the static and perfused conditions: Evidence for possible organ-to-organ interactions against exogenous stimuli. Mater. Sci. Eng. C.

[19] Du Y., Li N., Yang H., Luo C., Gong Y., Tong C., Gao Y., Lü S., Long M. (2017). Mimicking liver sinusoidal structures and functions using a 3D-configured microfluidic chip. Lab A Chip.

[20] Thomas R.J., Bhandari R., Barrett D.A., Bennett A.J., Fry J.R., Powe D., Thomson B.J., Shakesheff K.M. (2005). The Effect of Three-Dimensional Co-Culture of Hepatocytes and Hepatic Stellate Cells on Key Hepatocyte Functions in vitro. Cells Tissues Organs.

[21] Leung C.M., De Haan P., Ronaldson-Bouchard K., Kim G.-A., Ko J., Rho H.S., Chen Z., Habibovic P., Jeon N.L., Takayama S. (2022). A guide to the organ-on-a-chip. Nat. Rev. Methods Primers.

[22] Ma C., Peng Y., Li H., Chen W. (2021). Organ-on-a-Chip: A New Paradigm for Drug Development. Trends Pharmacol. Sci..

[23] Gough A., Soto-Gutierrez A., Vernetti L., Ebrahimkhani M.R., Stern A.M., Taylor D.L. (2021). Human biomimetic liver microphysiology systems in drug development and precision medicine. Nat. Rev. Gastroenterol. Hepatol..

[24] Ewart L., Apostolou A., Briggs S.A., Carman C.V., Chaff J.T., Heng A.R., Jadalannagari S., Janardhanan J., Jang K.-J., Joshipura S.R. (2022). Performance assessment and economic analysis of a human Liver-Chip for predictive toxicology. Commun. Med..

[25] Wisse E., Braet F., Luo D., De Zanger R., Jans D., Crabbe E., Vermoesen A. (1996). Structure and Function of Sinusoidal Lining Cells in the Liver. Toxicol. Pathol..

[26] Sanz-García C., Fernández-Iglesias A., Gracia-Sancho J., Arráez-Aybar L.A., Nevzorova Y.A., Cubero F.J. (2021). The Space of Disse: The Liver Hub in Health and Disease. Livers.

[27] Zong Y., Stanger B.Z. (2012). Molecular mechanisms of liver and bile duct development. WIREs Dev. Biol..

[28] Deguchi S., Takayama K. (2022). State-of-the-art liver disease research using liver-on-a-chip. Inflamm. Regen..

[29] Kietzmann T. (2017). Metabolic zonation of the liver: The oxygen gradient revisited. Redox Biol..

[30] Kusminski C.M., Scherer P.E. (2018). New zoning laws enforced by glucagon. Proc. Natl. Acad. Sci. USA.

[31] Adam M.P., Feldman J., Mirzaa G.M., Pagon R.A., Wallace S.E., Bean L.J., Gripp K.W., Amemiya A. (1993). GeneReviews^®^.

[32] Zhao M., Ma J., Li M., Zhang Y., Jiang B., Zhao X., Huai C., Shen L., Zhang N., He L. (2021). Cytochrome P450 Enzymes and Drug Metabolism in Humans. Int. J. Mol. Sci..

[33] Rendic S., Guengerich F.P. (2015). Survey of Human Oxidoreductases and Cytochrome P450 Enzymes Involved in the Metabolism of Xenobiotic and Natural Chemicals. Chem. Res. Toxicol..

[34] Testa B., Pedretti A., Vistoli G. (2012). Reactions and enzymes in the metabolism of drugs and other xenobiotics. Drug Discov. Today.

[35] Mao S., Gao D., Liu W., Wei H., Lin J.-M. (2012). Imitation of drug metabolism in human liver and cytotoxicity assay using a microfluidic device coupled to mass spectrometric detection. Lab A Chip.

[36] Deng J., Chen Z., Zhang X., Luo Y., Wu Z., Lu Y., Liu T., Zhao W., Lin B. (2019). A liver-chip-based alcoholic liver disease model featuring multi-non-parenchymal cells. Biomed. Microdevices.

[37] Banaeiyan A.A., Theobald J., Paukštyte J., Wölfl S., Adiels C.B., Goksör M. (2017). Design and fabrication of a scalable liver-lobule-on-a-chip microphysiological platform. Biofabrication.

[38] Tan K., Keegan P., Rogers M., Lu M., Gosset J.R., Charest J., Bale S.S. (2019). A high-throughput microfluidic microphysiological system (PREDICT-96) to recapitulate hepatocyte function in dynamic, re-circulating flow conditions. Lab A Chip.

[39] Cui J., Wang H.P., Shi Q., Sun T. (2021). Pulsed Microfluid Force-Based On-Chip Modular Fabrication for Liver Lobule-Like 3D Cellular Models. Cyborg Bionic Syst..

[40] Bhise N.S., Manoharan V., Massa S., Tamayol A., Ghaderi M., Miscuglio M., Lang Q., Shrike Zhang Y., Shin S.R., Calzone G. (2016). A liver-on-a-chip platform with bioprinted hepatic spheroids. Biofabrication.

[41] Lee S.-A., No D.Y., Kang E., Ju J., Kim D.-S., Lee S.-H. (2013). Spheroid-based three-dimensional liver-on-a-chip to investigate hepatocyte–hepatic stellate cell interactions and flow effects. Lab A Chip.

[42] Kulsharova G., Kurmangaliyeva A., Darbayeva E., Rojas-Solórzano L., Toxeitova G. (2021). Development of a Hybrid Polymer-Based Microfluidic Platform for Culturing Hepatocytes towards Liver-on-a-Chip Applications. Polymers.

[43] Kamei K., Yoshioka M., Terada S., Tokunaga Y., Chen Y. (2019). Three-dimensional cultured liver-on-a-Chip with mature hepatocyte-like cells derived from human pluripotent stem cells. Biomed. Microdevices.

[44] Ong L.J.Y., Chong L.H., Jin L., Singh P.K., Lee P.S., Yu H., Ananthanarayanan A., Leo H.L., Toh Y. (2017). A pump-free microfluidic 3D perfusion platform for the efficient differentiation of human hepatocyte-like cells. Biotech. Bioeng..

[45] Matsumoto S., Leclerc E., Maekawa T., Kinoshita H., Shinohara M., Komori K., Sakai Y., Fujii T. (2018). Integration of an oxygen sensor into a polydymethylsiloxane hepatic culture device for two-dimensional gradient characterization. Sens. Actuators B Chem..

[46] Deng J., Cong Y., Han X., Wei W., Lu Y., Liu T., Zhao W., Lin B., Luo Y., Zhang X. (2020). A liver-on-a-chip for hepatoprotective activity assessment. Biomicrofluidics.

[47] Rennert K., Steinborn S., Gröger M., Ungerböck B., Jank A.-M., Ehgartner J., Nietzsche S., Dinger J., Kiehntopf M., Funke H. (2015). A microfluidically perfused three dimensional human liver model. Biomaterials.

[48] Zhou Q., Patel D., Kwa T., Haque A., Matharu Z., Stybayeva G., Gao Y., Diehl A.M., Revzin A. (2015). Liver injury-on-a-chip: Microfluidic co-cultures with integrated biosensors for monitoring liver cell signaling during injury. Lab A Chip.

[49] Prodanov L., Jindal R., Bale S.S., Hegde M., McCarty W.J., Golberg I., Bhushan A., Yarmush M.L., Usta O.B. (2016). Long-term maintenance of a microfluidic 3D human liver sinusoid. Biotech. Bioeng..

[50] Hegde M., Jindal R., Bhushan A., Bale S.S., McCarty W.J., Golberg I., Usta O.B., Yarmush M.L. (2014). Dynamic interplay of flow and collagen stabilizes primary hepatocytes culture in a microfluidic platform. Lab A Chip.

[51] Lee H., Chae S., Kim J.Y., Han W., Kim J., Choi Y., Cho D.-W. (2019). Cell-printed 3D liver-on-a-chip possessing a liver microenvironment and biliary system. Biofabrication.

[52] Meng Q., Wang Y., Li Y., Shen C. (2021). Hydrogel microfluidic-based liver-on-a-chip: Mimicking the mass transfer and structural features of liver. Biotech. Bioeng..

[53] Delalat B., Cozzi C., Rasi Ghaemi S., Polito G., Kriel F.H., Michl T.D., Harding F.J., Priest C., Barillaro G., Voelcker N.H. (2018). Microengineered Bioartificial Liver Chip for Drug Toxicity Screening. Adv. Funct. Mater..

[54] Moya A., Ortega-Ribera M., Guimerà X., Sowade E., Zea M., Illa X., Ramon E., Villa R., Gracia-Sancho J., Gabriel G. (2018). Online oxygen monitoring using integrated inkjet-printed sensors in a liver-on-a-chip system. Lab A Chip.

[55] Bavli D., Prill S., Ezra E., Levy G., Cohen M., Vinken M., Vanfleteren J., Jaeger M., Nahmias Y. (2016). Real-time monitoring of metabolic function in liver-on-chip microdevices tracks the dynamics of mitochondrial dysfunction. Proc. Natl. Acad. Sci. USA.

[56] Du K., Li S., Li C., Li P., Miao C., Luo T., Qiu B., Ding W. (2021). Modeling nonalcoholic fatty liver disease on a liver lobule chip with dual blood supply. Acta Biomater..

[57] Janani G., Priya S., Dey S., Mandal B.B. (2022). Mimicking Native Liver Lobule Microarchitecture In Vitro with Parenchymal and Non-parenchymal Cells Using 3D Bioprinting for Drug Toxicity and Drug Screening Applications. ACS Appl. Mater. Interfaces.

[58] Ya S., Ding W., Li S., Du K., Zhang Y., Li C., Liu J., Li F., Li P., Luo T. (2021). On-Chip Construction of Liver Lobules with Self-Assembled Perfusable Hepatic Sinusoid Networks. ACS Appl. Mater. Interfaces.

[59] Ma C., Zhao L., Zhou E.-M., Xu J., Shen S., Wang J. (2016). On-Chip Construction of Liver Lobule-like Microtissue and Its Application for Adverse Drug Reaction Assay. Anal. Chem..

[60] Liu J., Feng C., Zhang M., Song F., Liu H. (2022). Design and Fabrication of a Liver-on-a-chip Reconstructing Tissue-tissue Interfaces. Front. Oncol..

[61] Domansky K., Inman W., Serdy J., Dash A., Lim M.H.M., Griffith L.G. (2010). Perfused multiwell plate for 3D liver tissue engineering. Lab A Chip.

[62] Weng Y., Chang S., Shih M., Tseng S., Lai C. (2017). Scaffold-Free Liver-On-A-Chip with Multiscale Organotypic Cultures. Adv. Mater..

[63] Busche M., Rabl D., Fischer J., Schmees C., Mayr T., Gebhardt R., Stelzle M. (2022). Continous, non-invasive monitoring of oxygen consumption in a parallelized microfluidic in vitro system provides novel insight into the response to nutrients and drugs of primary human hepatocytes. EXCLI J..

[64] Kamei K., Mashimo Y., Koyama Y., Fockenberg C., Nakashima M., Nakajima M., Li J., Chen Y. (2015). 3D printing of soft lithography mold for rapid production of polydimethylsiloxane-based microfluidic devices for cell stimulation with concentration gradients. Biomed. Microdevices.

[65] Lee G., Kim H., Park J.Y., Kim G., Han J., Chung S., Yang J.H., Jeon J.S., Woo D.-H., Han C. (2021). Generation of uniform liver spheroids from human pluripotent stem cells for imaging-based drug toxicity analysis. Biomaterials.

[66] Gori M., Simonelli M.C., Giannitelli S.M., Businaro L., Trombetta M., Rainer A. (2016). Investigating Nonalcoholic Fatty Liver Disease in a Liver-on-a-Chip Microfluidic Device. PLoS ONE.

[67] Jeon J., Lee S.H., Kim D., Sung J.H. (2021). In vitro hepatic steatosis model based on gut–liver-on-a-chip. Biotechnol. Progress..

[68] Chen W.L.K., Edington C., Suter E., Yu J., Velazquez J.J., Velazquez J.G., Shockley M., Large E.M., Venkataramanan R., Hughes D.J. (2017). Integrated gut/liver microphysiological systems elucidates inflammatory inter-tissue crosstalk. Biotech. Bioeng..

[69] Kanai H., Marushima H., Kimura N., Iwaki T., Saito M., Maehashi H., Shimizu K., Muto M., Masaki T., Ohkawa K. (2007). Extracorporeal Bioartificial Liver Using the Radial-flow Bioreactor in Treatment of Fatal Experimental Hepatic Encephalopathy. Artif. Organs.

[70] Ishii Y., Saito R., Marushima H., Ito R., Sakamoto T., Yanaga K. (2008). Hepatic reconstruction from fetal porcine liver cells using a radial flow bioreactor. World J. Gastroenterol..

[71] Kawada M., Nagamori S., Aizaki H., Fukaya K., Niiya M., Matsuura T., Sujino H., Hasumura S., Yashida H., Mizutani S. (1998). Massive culture of human liver cancer cells in a newly developed radial flow bioreactor system: Ultrafine structure of functionally enhanced hepatocarcinoma cell lines. In Vitro Cell. Dev. Biol.-Anim..

[72] Lorente S., Hautefeuille M., Sanchez-Cedillo A. (2020). The liver, a functionalized vascular structure. Sci. Rep..

[73] Boul M., Benzoubir N., Messina A., Ghasemi R., Mosbah I.B., Duclos-Vallée J.-C., Dubart-Kupperschmitt A., Le Pioufle B. (2021). A versatile microfluidic tool for the 3D culture of HepaRG cells seeded at various stages of differentiation. Sci. Rep..

[74] Zhdanov A.V., Ogurtsov V.I., Taylor C.T., Papkovsky D.B. (2010). Monitoring of cell oxygenation and responses to metabolic stimulation by intracellular oxygen sensing technique. Integr. Biol..

[75] Ghafoory S., Stengl C., Kopany S., Mayadag M., Mechtel N., Murphy B., Schattschneider S., Wilhelmi N., Wölfl S. (2022). Oxygen Gradient Induced in Microfluidic Chips Can Be Used as a Model for Liver Zonation. Cells.

[76] Kwon D., Choi G., Park S.-A., Cho S., Cho S., Ko S. (2022). Liver Acinus Dynamic Chip for Assessment of Drug-Induced Zonal Hepatotoxicity. Biosensors.

[77] Toh Y.-C., Lim T.C., Tai D., Xiao G., Van Noort D., Yu H. (2009). A microfluidic 3D hepatocyte chip for drug toxicity testing. Lab A Chip.

[78] LeCluyse E.L. (2001). Human hepatocyte culture systems for the in vitro evaluation of cytochrome P450 expression and regulation. Eur. J. Pharm. Sci..

[79] Burkhardt B., Martinez-Sanchez J.J., Bachmann A., Ladurner R., Nüssler A.K. (2014). Long-term culture of primary hepatocytes: New matrices and microfluidic devices. Hepatol. Int..

[80] Zheng Y., Ma L., Wu J., Wang Y., Meng X., Hu P., Liang Q., Xie Y., Luo G. (2022). Design and fabrication of an integrated 3D dynamic multicellular liver-on-a-chip and its application in hepatotoxicity screening. Talanta.

[81] Villeneuve J.-P., Pichette V. (2004). Cytochrome P450 and Liver Diseases. Curr. Drug Metab..

[82] Takemura A., Gong S., Sato T., Kawaguchi M., Sekine S., Kazuki Y., Horie T., Ito K. (2021). Evaluation of Parent- and Metabolite-Induced Mitochondrial Toxicities Using CYP-Introduced HepG2 cells. J. Pharm. Sci..

[83] Parrish J., Lim K.S., Baer K., Hooper G.J., Woodfield T.B.F. (2018). A 96-well microplate bioreactor platform supporting individual dual perfusion and high-throughput assessment of simple or biofabricated 3D tissue models. Lab A Chip.

[84] Lee S.Y., Sung J.H. (2018). Gut–liver on a chip toward an in vitro model of hepatic steatosis. Biotech. Bioeng..

[85] Skardal A., Murphy S.V., Devarasetty M., Mead I., Kang H.-W., Seol Y.-J., Shrike Zhang Y., Shin S.-R., Zhao L., Aleman J. (2017). Multi-tissue interactions in an integrated three-tissue organ-on-a-chip platform. Sci. Rep..

[86] Lee-Montiel F.T., George S.M., Gough A.H., Sharma A.D., Wu J., DeBiasio R., Vernetti L.A., Taylor D.L. (2017). Control of oxygen tension recapitulates zone-specific functions in human liver microphysiology systems. Exp. Biol. Med..

[87] Feaver R.E., Cole B.K., Lawson M.J., Hoang S.A., Marukian S., Blackman B.R., Figler R.A., Sanyal A.J., Wamhoff B.R., Dash A. (2016). Development of an in vitro human liver system for interrogating nonalcoholic steatohepatitis. JCI Insight.

[88] Li X., George S.M., Vernetti L., Gough A.H., Taylor D.L. (2018). A glass-based, continuously zonated and vascularized human liver acinus microphysiological system (vLAMPS) designed for experimental modeling of diseases and ADME/TOX. Lab A Chip.

[89] Foster A.J., Chouhan B., Regan S.L., Rollison H., Amberntsson S., Andersson L.C., Srivastava A., Darnell M., Cairns J., Lazic S.E. (2019). Integrated in vitro models for hepatic safety and metabolism: Evaluation of a human Liver-Chip and liver spheroid. Arch. Toxicol..

[90] Benzait Z., Tomsuk Ö., Ebrahimi A., Ghorbanpoor H., Özel C., Didarian R., Demir Cevizlidere B., Kaya M., Gur T., Gasimzade N. (2026). Liver-on-a-Chip (LoC) Models: Case Studies of Academic Platforms and Commercial Products. Mol. Pharm..

[91] Bhattacharjee N., Urrios A., Kang S., Folch A. (2016). The upcoming 3D-printing revolution in microfluidics. Lab A Chip.

[92] Lindner N., Blaeser A. (2022). Scalable Biofabrication: A Perspective on the Current State and Future Potentials of Process Automation in 3D-Bioprinting Applications. Front. Bioeng. Biotechnol..

[93] Brandenberg N., Hoehnel S., Kuttler F., Homicsko K., Ceroni C., Ringel T., Gjorevski N., Schwank G., Coukos G., Turcatti G. (2020). High-throughput automated organoid culture via stem-cell aggregation in microcavity arrays. Nat. Biomed. Eng..

[94] Zhang W., Li J., Zhou J., Rastogi A., Ma S. (2022). Translational organoid technology—The convergence of chemical, mechanical, and computational biology. Trends Biotechnol..

[95] Liang Y., Yoon J.-Y. (2021). In situ sensors for blood-brain barrier (BBB) on a chip. Sens. Actuators Rep..

[96] Li Q., Lin Z., Liu R., Tang X., Huang J., He Y., Sui X., Tian W., Shen H., Zhou H. (2023). Multimodal charting of molecular and functional cell states via in situ electro-sequencing. Cell.

[97] Yu S., Zhou Y., Sun Y., Wu S., Xu T., Chang Y., Bi S., Jiang L., Zhu J. (2021). Endogenous mRNA Triggered DNA-Au Nanomachine for In Situ Imaging and Targeted Multimodal Synergistic Cancer Therapy. Angew. Chem. Int. Ed..

[98] De Chiara F., Ferret-Miñana A., Ramón-Azcón J. (2021). The Synergy between Organ-on-a-Chip and Artificial Intelligence for the Study of NAFLD: From Basic Science to Clinical Research. Biomedicines.

[99] Wang Y., Wu D., Wu G., Wu J., Lu S., Lo J., He Y., Zhao C., Zhao X., Zhang H. (2020). Metastasis-on-a-chip mimicking the progression of kidney cancer in the liver for predicting treatment efficacy. Theranostics.

[100] Hassan S., Sebastian S., Maharjan S., Lesha A., Carpenter A., Liu X., Xie X., Livermore C., Zhang Y.S., Zarrinpar A. (2020). Liver-on-a-Chip Models of Fatty Liver Disease. Hepatology.

[101] Underhill G.H., Khetani S.R. (2019). Emerging trends in modeling human liver disease in vitro. APL Bioeng..

[102] Moradi E., Jalili-Firoozinezhad S., Solati-Hashjin M. (2020). Microfluidic organ-on-a-chip models of human liver tissue. Acta Biomater..

[103] Liu H., Zhang X., Wang Y., Zhang M., Wang P., Shang J., Li Z., Gong L., Xie X., Liu D. (2025). Standard: Human liver-on-a-chip. Cell Regen..

[104] Mugaanyi J., Huang J., Fang J., Musinguzi A., Lu C., Chen Z. (2025). Developments and Applications of Liver-on-a-Chip Technology—Current Status and Future Prospects. Biomedicines.

[105] Nawroth J.C., Petropolis D.B., Manatakis D.V., Maulana T.I., Burchett G., Schlünder K., Witt A., Shukla A., Kodella K., Ronxhi J. (2021). Modeling alcohol-associated liver disease in a human Liver-Chip. Cell Rep..

[106] Wang Y., Wang H., Deng P., Tao T., Liu H., Wu S., Chen W., Qin J. (2020). Modeling Human Nonalcoholic Fatty Liver Disease (NAFLD) with an Organoids-on-a-Chip System. ACS Biomater. Sci. Eng..

[107] Akahori Y., Kato H., Fujita T., Moriishi K., Tanaka Y., Watashi K., Imamura M., Chayama K., Wakita T., Hijikata M. (2020). Establishment of a novel hepatitis B virus culture system using immortalized human hepatocytes. Sci. Rep..

[108] Natarajan V., Simoneau C.R., Erickson A.L., Meyers N.L., Baron J.L., Cooper S., McDevitt T.C., Ott M. (2022). Modelling T-cell immunity against hepatitis C virus with liver organoids in a microfluidic coculture system. Open Biol..

[109] Ortega-Prieto A.M., Skelton J.K., Wai S.N., Large E., Lussignol M., Vizcay-Barrena G., Hughes D., Fleck R.A., Thursz M., Catanese M.T. (2018). 3D microfluidic liver cultures as a physiological preclinical tool for hepatitis B virus infection. Nat. Commun..

[110] Lima A.M., Feitor J.F., Ferreira V.G., Almeida M.B., Brazaca L.C., Cardoso D.R., Carrilho E., Crespilho F.N. (2023). “Pandemics-on-a-Chip”: Organ-on-a-Chip Models for Studying Viral Infections. COVID-19 Metabolomics and Diagnosis.

[111] Sodunke T.R., Bouchard M.J., Noh H. (2008). (Moses) Microfluidic platform for hepatitis B viral replication study. Biomed. Microdevices.

[112] Kang Y., Rawat S., Duchemin N., Bouchard M., Noh M. (2017). Human Liver Sinusoid on a Chip for Hepatitis B Virus Replication Study. Micromachines.

[113] Liu H., Yin G., Kohlhepp M.S., Schumacher F., Hundertmark J., Hassan M.I.A., Heymann F., Puengel T., Kleuser B., Mosig A.S. (2024). Dissecting Acute Drug-Induced Hepatotoxicity and Therapeutic Responses of Steatotic Liver Disease Using Primary Mouse Liver and Blood Cells in a Liver-On-A-Chip Model. Adv. Sci..

[114] Rezvani M., Vallier L., Guillot A. (2023). Modeling Nonalcoholic Fatty Liver Disease in the Dish Using Human-Specific Platforms: Strategies and Limitations. Cell. Mol. Gastroenterol. Hepatol..

[115] Satta S., Rockwood S.J., Wang K., Wang S., Mozneb M., Arzt M., Hsiai T.K., Sharma A. (2023). Microfluidic Organ-Chips and Stem Cell Models in the Fight Against COVID-19. Circ. Res..

[116] Deguchi S., Kosugi K., Hashimoto R., Sakamoto A., Yamamoto M., Krol R.P., Gee P., Negoro R., Noda T., Yamamoto T. (2023). Elucidation of the liver pathophysiology of COVID-19 patients using liver-on-a-chips. PNAS Nexus.

[117] Negi V., Gavlock D., Miedel M.T., Lee J.K., Shun T., Gough A., Vernetti L., Stern A.M., Taylor D.L., Yechoor V.K. (2023). Modeling mechanisms underlying differential inflammatory responses to COVID-19 in type 2 diabetes using a patient-derived microphysiological organ-on-a-chip system. Lab A Chip.

